# Gene Therapy Today and Tomorrow

**DOI:** 10.3390/diseases7020037

**Published:** 2019-04-28

**Authors:** Kenneth Lundstrom

**Affiliations:** PanTherapeutics, CH 1095 Lutry, Switzerland; lundstromkenneth@gmail.com; Tel.: +41-79-776-6351

In the wake of a breakthrough in biotechnology providing realistic application of recombinant expressed proteins as drugs in the 1990s, gene therapy emerged as the potential approach for providing medicines of the future. Plenty of excitement of the seemingly unlimited opportunities of gene therapy resulted in the establishment of numerous preclinical and clinical trials for a variety of human diseases using both non-viral and viral vectors. Unfortunately, in the middle of all the excitement, the gene therapy community was hit by a couple of serious set-backs, when a young patient died in an adenovirus-based clinical trial on the none-life threatening disease ornithine carboxylase [1]. Furthermore, chromosomal integration of a retrovirus-delivered gene into the *LMO2* proto-oncogene resulted in leukemia in some children successfully treated for severe combined immunodeficiency (SCID) [2,3]. Obviously, these events resulted in serious skepticism and decreased funding of gene therapy initiatives with significant withdrawal of support from the pharmaceutical and biotech industry. Although the set-backs were somber and the whole field of gene therapy suffered, it also had some positive effects much to the gratitude of committed gene therapists. Serious contributions to the engineering of improved vectors related to delivery and safety have significantly elevated the quality of clinical trials. The renaissance in gene therapy has seen major development of both non-viral and viral vectors and accelerated preclinical studies and clinical trials. It is therefore timely to address the progress in gene therapy through a special issue presenting reviews on non-viral and viral vectors including relevant updates on applications on herpes simplex virus (HSV) and adeno-associated virus (AAV) vectors.

## 1. Non-Viral Vectors

In the context of non-viral gene delivery, Hidai and Kitano discuss the problems related to delivery [4]. In addition to non-viral transfection methods such as microinjection, electroporation, and encapsulation of nanoparticles, direct transfer of DNA to the cell nucleus by nucleofection has proven efficient [5]. However, to a large extent, viral vectors have in comparison to non-viral vectors demonstrated 10 times to 1000 times higher efficacy of gene transfer [6,7]. Clearly, the areas to focus on for improved non-viral vector efficiency relates to the steps between DNA uptake and transcription. Moreover, the high safety levels and low production costs are attractive features for non-viral vector-based gene therapy. Hidai and Kitano cover the application of non-viral vectors for gene therapy of cancer and other diseases [4]. In this context, minicircle DNA has been demonstrated to extend gene expression, which improved the wound healing process in a diabetic mouse model [8,9]. Moreover, introduction of the hepatic locus control regions has contributed to increase and stabilization of hepatic factor IX gene expression in vivo, although not at comparable levels achieved with genome-integrated viral vectors [10]. Despite the shortcomings with non-viral delivery improved cardiac function was obtained in rats with myocardial infarction after delivery of naked DNA coding for the stromal cell derived factor-1, and in patients with ischemic cardiomyopathy in a phase I clinical trial [11]. Related to cancer therapy, the polymer-encapsulated DNA vector expressing sodium iodide transporter (NIS) in combination with radioiodine therapy showed delayed tumor growth in syngeneic A/J mice [12]. In another study, plasmid DNA encoding the p53 tumor suppressor gene encapsulated in polymeric nanoparticles provided significant reduction in tumor growth and prolonged survival of mice with tumor xenografts [13]. Similar effects were detected after intravenous administration, but not at the same extent. Non-viral vectors have also been subjected to combination therapy for spontaneous melanoma in dogs with chemotherapy and cytotherapy, which induced tumor regression and pronounced immune cell filtration [14]. Moreover, combination therapy provided controlled tumor growth by delaying or preventing distant metastasis. Similarly, treatment with ganciclovir, interleukin-2 and DNA-based expression of human granulocyte macrophage colony stimulating factor (GM-CSF) induced local antitumor activity in dogs with osteosarcoma, and prevented or delayed local relapse, regional and distant metastases [15]. In summary, non-viral vectors have proven useful for local injections and repeated administration. They have also been confirmed to be safe and can be produced at reasonable costs.

## 2. Viral Vectors

Concerning viral vectors, the field has experienced an unprecedented progress related to delivery and safety issues as described in the review on Viral Vectors in Gene Therapy by Lundstrom [16]. Although adenoviruses [17] and retroviruses [18] have been by tradition most frequently used for gene therapy applications, the selection of viral vectors is quite impressive. For instance, adeno-associated virus (AAV) [19] and herpes simplex virus (HSV) [20] are presented in two separate reviews in this special issue. Moreover, self-amplifying RNA viruses such as alphaviruses, flaviviruses, rhabdoviruses and measles viruses have been engineered for gene therapy applications [21]. The self-amplifying nature of these viruses permits direct high capacity RNA replication in the cytoplasm and in the case of the positive-strand RNA alphaviruses and flaviviruses direct translation providing rapid high-level transient gene expression. On the other hand, the negative-strand RNA rhabdoviruses and measles viruses require an intermediate RNA template for translation [21]. Additionally, the ssRNA paramyxovirus Newcastle disease virus (NDV) replicates uniquely in tumor cells making it an attractive vector for cancer therapy [22]. Oncolytic properties have also been related to ssRNA Coxsackieviruses belonging to Picornaviridae [23]. An attractive alternative for virus-based gene therapy is represented by poxviruses, and particularly engineered vaccinia viruses replicating in tumor cells [24]. Although retroviruses have been commonly used, the lack of susceptibility of non-dividing cells has to some extent moved the focus to lentiviruses [25].

Different viral vectors have been subjected to numerous preclinical studies with a strong emphasis on cancer although other indications have been targeted too [16]. In this context, oncolytic adenoviruses have been subjected to studies on breast cancer [26], pancreatic cancer [27] and glioma [28] resulting in tumor regression. AAV vectors expressing methyl CpG protein 2 (MeCP2) and factor VIII, respectively, have been evaluated in a mouse model for the Rett Syndrome (RTT) resulting in prolonged survival in mice [29] and hemophilia, providing reduced muscular degeneration [30]. Moreover, oncolytic HSV showed tumor growth inhibition in a mouse colon tumor model [31]. Retroviral replicating vector (RRV) expressing cytosine deaminase (CD) demonstrated prolonged survival in a mouse glioma model after combined therapy with 5-fluorocytosine (5-FC) [32]. Related to lentiviruses, studies have been conducted with HIV vectors expressing small interfering RNA (siRNA) and short hairpin RNA (shRNA) resulting in reduced neurodegeneration in a mouse Alzheimer’s disease model [33] and inhibition of HIV infection [34], respectively. Alphaviruses, flaviviruses, rhabdoviruses and measles viruses have all been subjected to preclinical studies in animal tumor models [21]. Particularly, alphavirus vectors have been utilized as naked RNA replicons, recombinant particles and layered DNA/RNA vectors [35]. In addition to expression of toxic and anticancer genes, and immunostimulatory antigens, introduction of micro-RNA (miRNA) sequences into the replication-proficient SFV4 vector resulted in glioma targeting, limited virus spread in the CNS and significantly extended survival rates in BALB/c mice [36]. Furthermore, the naturally occurring oncolytic alphavirus M1 showed active tumor killing and oncolytic activity in a mouse liver tumor model [37]. Related to flaviviruses, intratumoral administration of Kunjin-GM-CSF vectors cured more than half of the mouse with CT26 colon tumor xenografts [38]. Similarly, an oncolytic vesicular stomatitis virus (VSV), a rhabdovirus, expressing human mucin 1 (MUC1) generated a significant reduction of tumor growth in mice implanted with pancreatic ductal adenocarcinoma xenografts [39]. Oncolytic measles viruses [40] and Newcastle disease virus (NDV) [41] also have demonstrated enhanced tumor killing and suppression of tumor growth. Finally, both Coxsackieviruses and vaccinia viruses have proven efficient in providing protection from ischemic necrosis [42] and tumor regression [43]. Furthermore, a combination of vaccinia-based NIS expression with radiotherapy showed superior tumor regression and improved survival rates in comparison to individual treatments [44].

In the context of clinical trials, both AAV [45] and lentiviruses [46] have been subjected to studies in hemophilia patients with some promising results of a cure. Moreover, oncolytic HSV vectors have been subjected to clinical trials for patients with recurrent breast cancer, head and neck cancer, unresectable pancreatic cancer, refractory superficial cancer and melanoma [47]. Among retroviruses, Toca 511 has been successfully applied in a phase I multicenter trial for recurrent or progressive high-grade glioma [48] and more recently in a phase II/III trial [49], while gammaretroviral vectors have been used for a phase I/II trial in patients with chronic granulomatous disease [50]. Alphaviruses have been subjected to few clinical trials, mainly applying VEE particles expressing prostate specific membrane antigen (PSMA) in a phase I trial in patients with castration resistant prostate cancer [51]. Moreover, a phase I trial in CMV-seronegative volunteers were immunized with VEE vectors expressing CMV fusion proteins [52]. Both studies elicited neutralizing antibodies albeit at low levels. In another phase I study, a liposome-encapsulated SFV vector expressing IL-12, provided safe administration and five-fold transient increase in IL-12 plasma levels in melanoma and kidney carcinoma patients [53]. Related to measles virus, MV-NIS was intravenously administered to patients with relapsed, refractory myeloma and despite not reaching a maximum tolerated dose (MTD) provided a complete response in one patient [54]. NDV vectors have been evaluated in several clinical trials providing long-term survival in a phase II trials in patients with ovarian, stomach and pancreatic cancer [55] and progression-free survival in a phase I trial in patients with solid tumors [56]. Coxsackieviruses have been subjected to a phase I/II trial in melanoma patients showing good tolerance, and antitumor activity, which could be further enhanced by checkpoint blockade-based combination therapy [57]. Similarly, co-administration of Coxsackievirus CVA21 and pembrilizumab resulted in a best overall response rate of 60% and stable disease in 27% in a phase Ib trial in melanoma patients [58]. Furthermore, oncolytic vaccinia viruses showed safe administration in a phase I clinical trial in patients with refractory advanced colorectal or other solid cancers [59]. Moreover, intratumoral injection of PANVAC-VF, a priming dose of vaccinia virus and booster dose of fowlpox virus expressing CEA, MUC-1 and a triad of costimulatory molecules (TRICOM), has been evaluated in patients with advanced pancreatic cancer with promising results [60].

The special issue includes a more detailed insight into HSV vectors for applications in the CNS [20]. The extension of life-expectancy has significantly enhanced the occurrence of neurodegenerative diseases affecting the quality of life especially in the aging population. For this reason, there is a more urgent need to develop novel improved approaches to treat neurodegenerative disorders. One characteristic feature of HSV vectors is their suitability for transfer and long-term expression of large and multiple genes in neurons and thereby comprising an attractive tool for gene delivery and genetic interventions. Improved HSV vectors deficient in expression of HSV IE genes have demonstrated prevention of the induction of inflammation, neuronal damage and perturbation of nerve cell function [61]. Moreover, ICPO^+^ vectors with promoter systems for regulated transgene expression in sensory neurons have been engineered for chronic pain treatment [62]. In attempts to achieve selective transduction of tumor cells, HSV vectors containing a single-chain antibody (scFv) to HER-2, commonly overexpressed in breast and ovarian cancers, demonstrated utilization of HER-2 as the sole receptor in vivo when introduced at the N-terminus of the hG glycoprotein. The highly cancer-specific targeting and replication in tumor cells will allow systemic administration. Another approach for application of HSV has been the use of HSV amplicon vectors [63]. These minimal HSV vectors possess an impressive packaging capacity of 150 kb, but require a helper virus for packaging and remain extrachromosomal with no risk of insertional mutagenesis [64]. Related to the future applications of HSV vectors, a combination of gene therapy and gene editing is foreseen including homologous repair of defective genes [20].

In the review on AAV, Rabinowitz and co-workers discuss the host immune response related to viral gene delivery [19]. Although AAV vectors are characterized by low pathogenicity and toxicity, one limitation relates to immune responses triggered by repeated AAV administration, which has compromised gene transfer efficacy in several clinical trials [65,66]. In this context, it was determined that an AAV capsid-specific CD8^+^ cytotoxic T cell response was the likely cause of decline in factor IX (F IX) expression in patients [67]. Moreover, it has been demonstrated that the capsid-specific CD8^+^ T cell population recognized and destroyed AAV-transduced cells. Likewise, limited transgene expression was observed in limb girdle muscular dystrophy (LGMD) patients intramuscularly injected with AAV1 expressing γ-sarcoglycan and AAV expressing the mini-dystrophin gene administered to Duchenne muscular dystrophy (DMD) patients [68]. To address the immunogenicity of AAV capsids, insertional mutagenesis showed flexibility and reduced binding and neutralization [69]. In another approach, specific inhibitors of the epidermal growth factor receptor protein tyrosine kinase (EGFR-PTK) reduced transduction inhibition of AAV2 [70]. Furthermore, a mutagenesis approach of surface displayed tyrosine and phenylalainine residues resulted in enhanced transduction efficiency both in vitro and in vivo. The availability of cryo-EM structures of AAV has further supported optimization of transduction efficacy and reduction of immunogenicity by the visualization of protein binding interactions between AAV serotypes and antibodies [71]. Another approach has been to introduce mutations into the AAV structural genes by error prone PCR and by generating AAV particles with chimeric capsids, which show 100-fold higher resistance to neutralizing antibodies [72]. Moreover, family shuffling of multiple AAV serotypes generated chimeric AAV-DJ particles, which when administered three weeks after AAV2 injection resulted in no cross reactivity [73].

## 3. Specific Applications

The special issue on Gene Therapy is also honored to include two research articles with practical implementations of gene therapy. Related to inflammatory-mediated reactions contributing to various dermatological disorders, Al-Shobaili and Rasheed have evaluated the potential of interleukin-32 (IL-32) and its isoforms in the contribution to the pathogenesis of psoriasis [74]. Patients with chronic plaque psoriasis showed higher IL-32 mRNA levels in peripheral blood mononuclear cells (PBMCs) compared to healthy volunteers. Determination of IL-32 isoform mRNA levels demonstrated overexpression of all isoforms in psoriasis patients. Particularly, expression of the IL-32α isoform mRNA was significantly higher than other isoform mRNA levels in psoriasis patients. This novel association of IL-32 and its isoforms in PBMCs and psoriasis provides potential approaches for gene therapy applications by targeting IL-32.

The other research article relates to the p53 tumor suppressor gene levels in patients with chronic myeloid leukemia (CML) [75]. In this study, the differential effect of two tyrosine kinase inhibitors, imatinib and nilotinib on p53 gene levels in serum of CML patients was investigated. Imatinib inhibits the BCR-ABL tyrosine kinase by induction of apoptosis and has proven efficacy in diseases such as mastocytosis, myelodysplastic syndrome and CML [76]. However, it causes side effects including pancytopenia, heart failure and edema. In contrast, nilotinib has demonstrated 10 to 30-fold potency in comparison to imatinib and despite such side effects as nausea, headache and muscle pain, it has been mainly used for the treatment of imatinib-resistant CML [77]. In comparison to healthy controls, CML patients showed significantly higher serum levels of p53. Moreover, patients treated with nilotinib revealed higher p53 levels than those treated with imatinib. These findings has contributed to the understanding of the function of the p53 tumor suppressor gene and should support future gene therapy initiatives.

## 4. Conclusions and Future Aspects

During the last five years gene therapy has experienced some substantial progress for large numbers of indications [78]. Among the 3000 clinical trials conducted or currently in progress, most trials (64.6%) have focused on cancer. The other indications comprise of monogenic (10.5%), infectious (7.4%) and cardiovascular diseases (7.4%). As viral vectors have been used in nearly 70% of the trials much of the efforts have been dedicated to issues related to the delivery and safety of engineered vectors.

Concerning nonviral-based gene therapy, the strength relates to safety and cost issues. In comparison to drugs, physiologically active substances are more efficient and safer than novel chemicals and plasmid DNA manufacturing is relatively inexpensive [4]. Moreover, the stability of DNA facilitates transportation and storage, which has been confirmed by rehydration of lyophilized polycation-DNA complexes [79]. A crucial indication of current achievements in viral-based gene therapy is the success of approved drugs. Oncolytic adenoviruses expressing the p53 tumor suppressor gene (Gendicine^TM^) [80] and AdH101 with an E1b-55K deletion [81] have been approved for cancers with p53 mutations and head and neck cancer, respectively. Additionally, the second-generation oncolytic HSV-GM-CSF was approved in the US and Europe for melanoma treatment [82]. Although the AAV-based drug Glybera was approved for treatment of lipoprotein lipase deficiency, the high costs and limited demand of therapy for this rare disease resulted in its withdrawal from the market [83]. For this reason, one challenge relates to development of funding mechanisms, which are affordable within healthcare budgets allowing sustainable reimbursement possibilities [84]. Moreover, most encouragingly, several drugs such as the oncolytic vaccinia virus JX-594 (pexastimogene devacirrepvec) for hepatocellular carcinoma [85], Ad CG0070 expressing GM-CSF for bladder cancer [86], and reovirus-based pelareorep (Reolysin^®^) [87] for head and neck cancer should reach the market soon.

Both viral and non-viral vector engineering plays an important role in the development of novel improved delivery systems. In this context, various oncolytic viruses, including self-amplifying RNA viruses, have proven efficient in both vaccine and gene therapy approaches [16,21,35]. Moreover, engineering less cytotoxic HSV vectors for brain delivery to treat neurological disorders will further enhance the potential in gene therapy [20]. Recent progress in reducing immune responses towards AAV vectors will also increase the application range of these vectors for gene therapy [19]. Areas of interest also relates to increased applications of RNA interference through viral-based siRNA, shRNA and miRNA approaches for targeting various diseases [88]. Similarly, gene manipulation methods including CRISPR technology are attractive alternative approaches for disease treatment in the future.
